# Retraction: MCM5 aggravates the HDAC1-mediated malignant progression of lung cancer

**DOI:** 10.3389/fcell.2026.1910805

**Published:** 2026-06-23

**Authors:** Frontiers Editorial Office

The journal retracts the 02 August 2021 article cited above.

Following publication, concerns were raised regarding the integrity of the images in the published figures. Image duplication concerns were identified in Figures 2E, 4C.

The authors failed to provide a satisfactory explanation during the investigation, which was conducted in accordance with Frontiers’ policies. As a result, the data and conclusions of the article have been deemed unreliable and the article has been retracted.

This retraction was approved by the Chief Editors of Frontiers in Cell and Developmental Biology and the Chief Executive Editor of Frontiers. The authors contacted the journal to request this retraction.

Frontiers would like to thank the concerned readers who contacted us regarding the published article.

